# Development of a Polyethylene Glycol/Polymethyl Methacrylate-Based Binder System for a Borosilicate Glass Filler Suitable for Injection Molding

**DOI:** 10.3390/ma17061396

**Published:** 2024-03-19

**Authors:** Martin Zürn, Annika Schrage, Steffen Antusch, Nicole Bohn, Peter Holzer, Thomas Hanemann

**Affiliations:** 1 Institute for Applied Materials, Karlsruhe Institute of Technology, Hermann-von-Helmholtz-Platz 1, 76344 Eggenstein-Leopoldshafen, Germany; martin.zuern@kit.edu (M.Z.); annika.schrage@student.kit.edu (A.S.); steffen.antusch@kit.edu (S.A.); nicole.bohn@kit.edu (N.B.); peter.holzer@kit.edu (P.H.); 2 Department of Microsystems Engineering, University of Freiburg, Georges-Köhler-Allee 102, 79110 Freiburg, Germany

**Keywords:** glass injection molding, borosilicate glass molding, PEG/PMMA-based binder

## Abstract

Powder injection molding is an established, cost effective and often near-net-shape mass production process for metal or ceramic parts with complex geometries. This paper deals with the extension of the powder injection molding process chain towards the usage of a commercially available borosilicate glass and the realization of glass compounds with huge densities. The whole process chain consists of the individual steps of compounding, molding, debinding, and sintering. The first part, namely, the search for a suitable feedstock composition with a very high solid load and reliable molding properties, is mandatory for the successful manufacture of a dense glass part. The most prominent feature is the binder composition and the related comprehensive rheological characterization. In this work, a binder system consisting of polyethylene glycol and polymethylmethacrylate with stearic acid as a surfactant was selected and its suitability for glass injection molding was evaluated. The influence of all feedstock components on processing and of the process steps on the final sintered part was investigated for sintered glass parts with densities around 99% of the theoretical value.

## 1. Introduction

Inorganic glass has evolved over time from a building and packaging substance to an increasingly important high-tech material. This includes, for example, use of fiber optics in information technology. In addition, glass is playing an increasingly important role in the areas of health and energy production. Glasses can be adapted to almost any potential application due to the almost infinite possibilities of glass composition [1,2]. A significant disadvantage of the current glass processing methods is that all shaping processes happen in the molten state and require an enormous amount of energy [3,4]. One promising method to reduce the required energy is via the use of powder technology replication methods like the established injection molding, which was originally invented for the shaping of thermoplastics but whose use through the years has extended to polymer matrix composites. Injection molding allows the production of plastic components with high dimensional accuracy and complex geometry [5,6]. Over the course of time, this process has been further extended to include the material range of ceramics and metals after thermal post-processing by powder injection molding (PIM) [7,8,9,10,11,12]. The abbreviation PIM represents a process chain, where the metal or ceramic filler is embedded in a thermoplastic matrix, called a binder, and molded. After removal of the binder (debinding), the “shaped” powder is sintered to obtain the final metallic or ceramic component. Nowadays, the PIM process has a particular significance as a manufacturing technology for large quantities of metal and ceramic components with high geometric accuracy [13]. In contrast to “classic” liquid glass processing, the sintering process is carried out at a temperature of only 60–70% of the absolute melting temperature [8]. Quite surprisingly, glass injection molding (GIM) is still in its infancy. There are currently only a few publications on glass injection molding [14,15,16,17]. Mader et al. used a pure, fused silica glass with a binder system consisting of the partially water-soluble polyethylene glycol (PEG) and polyvinyl butyral (PVB) for injection molding [14]. They achieved excellent part properties, but the used initial nanosized silica filler and the applied feedstock preparation are not suitable for mass fabrication due to the intermediate wet processing, causing elevated costs [14]. Hidalgo et al. used recycled glass from food packaging [15]. For this purpose, a binder system of low-density polyethylene (LDPE), paraffin wax (PW), and stearic acid (SA), which is commonly applied in ceramic injection molding (CIM), was used [15]. They investigated feedstocks with a glass load between 55 and 70%, but phase separation occurred at loadings > 65%. After processing, they achieved density values around 90% of the theoretical density, which is quite low compared to ceramics like alumina. Sample transparency could not be achieved [15]. Giassi et al. investigated the feedstock formation and injection molding of a glass–ceramic filler system. The binder consisted of polypropylene, wax, and SA as a surfactant. The solid load varied between 50 and 60 Vol%. Finally, they achieved density values of 97% of the theoretical density [16]. Enriquez and coworkers also researched the powder injection molding of glass ceramics in a high-density polyethylene (HDPE)/wax/SA binder [17]. The applied solid content was 45–70 Vol%. After processing, they obtained a density around 97% of the theoretical value [17]. In [15,16,17], wax was used as the binder component, which must be removed after shaping, with hexane as the solvent in a liquid pre-debinding step. However, the use of hexane should be avoided due to a pronounced lack of sustainability and serious health issues.

For ceramics and metals, several binder systems are described in the literature, e.g., the mixture of polyethylene and wax in combination with SA as a surfactant has been widely used in PIM [18,19,20]. In addition, various binders based on partially water-soluble polymers such as PEG have been applied for environmental reasons, avoiding the abovementioned problematic hexane removal issue. A wide variety of materials have already been successfully molded with binder systems consisting of PEG/PVB or PEG and polymethyl methacrylate (PMMA) [21,22,23,24,25]. A quite recent overview of powder–binder systems used in injection molding can be found in [26]. In general, and as stated above, wax as the binder component should be substituted, e.g., by water-soluble components like PEG, to enable a more environmentally friendly liquid debinding using water.

With respect to the increasing relevance of sustainable material selection and processing, this paper describes a glass feedstock consisting of a commercial borosilicate glass with a partially water-soluble binder containing PEG as the low molecular mass polymer, PMMA as the large molecular mass polymer, and SA as the surfactant. This development, among others, is targeted to achieve higher sinter densities than the 97% described in the literature. This binder selection follows the previous work in PIM and ceramic or metal part realization by powder-based material extrusion (MEX) additive manufacturing [27,28]. The PMMA serves as the backbone polymer, which gives the component the necessary mechanical stability after molding (also denoted as green body stability). The water-soluble PEG reduces melt viscosity (plasticizer), enabling PIM of the glass filler-containing feedstock. The SA serves as the dispersant and ensures good wetting of the glass filler by the organic binder polymers. SA also acts as an agent promoting release from the metal mold inserts during demolding. The borosilicate glass powder applied here has already been used in PIM [29] and in additive manufacturing [30] applying commercial binders. This early work will now be extended towards a systematic binder development with variable solid loads, average molar masses of the backbone polymer PMMA, as well as of the water-soluble PEG, PEG/PMMA ratios, and SA amounts. In all cases, the impact of this parameter variation on the rheological feedstock properties, on the molding behavior, as well as on the final sintered part properties will be discussed in detail, enabling the determination of a clear process parameter–properties relationship for a robust GIM process chain. The process chain consists of the following individual steps:

(a) feedstock preparation (compounding), including material selection;
(b) comprehensive rheological characterization with respect to feedstock composition, shear rate, and temperature;
(c) debinding, covering the steps of liquid pre-debinding and thermal debinding;
(d) sintering, including characterization of the sintered part.

The most important process step is compounding, because the solid load must be as high as possible to obtain a final dense part, which may result in a high feedstock viscosity. For complete mold filling, viscosity should be small, which is accompanied by a lower solid load. These contractionary requirements can only be solved by a careful binder material selection and comprehensive evaluation of the binder amount. Feedstock viscosity is mostly determined by the solid load or, in more detail, by the specific surface area (SSA) of the filler used. The SSA is the interface between the inorganic solid and the organic polymer matrix [31]. In the case of micro-sized ceramic fillers, such as Al_2_O_3_ or ZrO_2_ with typical SSA values around 6–12 m^2^/g, a solid loading of around 45–55 Vol% ensures a good molding behavior [18,27,31]. Use of micro-sized metal fillers with SSA values significantly below 1 m^2^/g enables higher solid loads between 60 and 65 Vol% [28]. The final part quality is limited by the necessity of finding a suitable feedstock meeting the above-mentioned requirements. More details on the selected materials and the process parameters will be given in the following sections.

## 2. Materials and Methods

### 2.1. Material Selection

**Figure 1:**
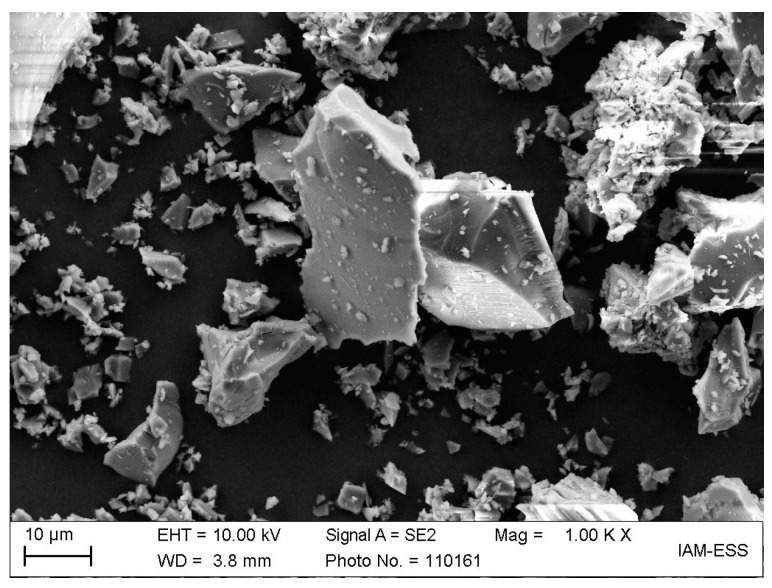
SEM of the applied borosilicate glass (Schott, Mainz, Germany) with an irregular morphology.

In the continuation of research work reported in the literature [29,30], a commercial, irregularly shaped glass powder (Schott 8250) with a density of 2.3 g/cm^3^, an average particle size (d_50_) of 10.2 µm, and an SSA of 1.6 m^2^/g was used. The irregular morphology is obvious from Figure 1.

A series of different new glass feedstocks were developed, containing mixtures of the water-soluble PEG, PMMA, and SA. The amount of SA was calculated in relation to the glass filler-specific surface area (mg/m^2^). Increasing SA amounts were compensated by an accordingly reduced PMMA fraction. In addition, different PMMA and PEG types with different molecular masses were investigated (Table 1). To evaluate the impact of the feedstock composition on processing conditions as well as on the final glass part properties, the solid powder load, the average molar mass M_W_ of PEG and PMMA, the ratio of PEG and PMMA in the binder, and the SA concentration were varied systematically. Previous work that dealt with the development of feedstock containing Ti6Al4V powder as a filler focused on PEG with different M_w_, G7E PMMA, different PEG/PMMA ratios, and SA as surfactant [28]. The results obtained were considered when investigating the feedstock composition; details can be found in the subsections of Section 3.

### 2.2. Compounding and Rheological Characterization

Prior to any shaping or replication, a set of basic processing and feedstock characterization steps are required. As a standard method, compounding was performed in a torque recording compounder (W50 EHT; Brabender, Duisburg, Germany). It allows for in-line torque recording during mixing to visualize the compounding progress at a given temperature over time. For the feedstocks based on PMMA G7E, G77, and Sigma 120k, a mixing temperature of 160 °C was set. For better comparison, a temperature of 125 °C only was necessary for the Sigma 15k feedstock due to the significantly lower molecular mass and, as a consequence, lower melt viscosity. All feedstocks were mixed for 1 h with a mixing speed of 30 rpm. As in previous work, e.g., in [28], the mixing chamber volume was 45 cm^3^. Successful compounding as a function of the feedstock composition can be obtained directly from the torque vs. time curve by considering the final equilibrium torque value. In this way, the limits of this technique can also be derived [31]. After compounding, the feedstocks were characterized using a high-pressure capillary rheometer at 170 °C for the high-molecular-mass PMMA-based systems and at 120–140 °C for the Sigma 15k-containing mixtures, again with the exception of feedstock 4 for better comparison. These temperatures were almost identical with those used in injection molding. The rheological characterization was performed with a high-pressure capillary viscosimeter (Rheograph 25; Göttfert GmbH, Buchen, Germany). The used capillary had a diameter of 1 mm and a length of 30 mm. The shear rate varied between 10 and 3500 s^−1^. The rheological data obtained allowed conclusions to be drawn as to whether the feedstock was homogeneous and suitable for injection molding.

### 2.3. Glass Injection Molding

For tests, green bodies with a diameter of 10 mm and a thickness of 2 mm were fabricated from all feedstocks using an injection molding machine designed for small and micro-sized parts needing only small amounts of feedstock (Microsystem 50; Battenfeld, Kottingbrunn, Austria). Depending on the feedstock composition, different molding parameters were chosen. The necessary dimensional stability was guaranteed by a holding pressure during cooling prior to demolding.

### 2.4. Debinding

The green bodies were debinded in two different ways. First, the binder components were removed using a thermal treatment at elevated temperatures. Second, liquid pre-debinding in de-ionized water was combined with a subsequent thermal treatment. Liquid pre-debinding allows for PEG recycling and further usage. For complete PEG removal, the necessary liquid debinding time and temperature were varied. In the case of thermal debinding, the focus was placed on the generation of defect-free test structures, which requires small heating rates. The experiments were performed using a Carbolite HT/28 (Carbolite, Neuhausen, Germany) chamber oven. The green bodies were placed onto alumina sintering plates. The selected temperatures, heating rates, and dwell times for PEG, PMMA, and SA were taken from [32]. After thermal debinding, the test structures are called brown bodies by convention.

### 2.5. Sintering and Further Densification by Hot Isostatic Pressing (HIP)

The debinded brown bodies were sintered under two different conditions. First, standard atmospheric conditions were chosen when applying a Carbolite HTF17/5 (Carbolite, Neuhausen, Germany) chamber oven. Second, sintering was carried out in a vacuum oven MUT ISO 350/300–2400 W (MUT–Jena, Jena, Germany). In all cases, the test structures were placed onto alumina sintering plates. Some sintered glass samples were additionally treated by hot isostatic pressing (HIP 3000; Dieffenbacher, Eppingen, Germany) for further densification by void removal in the case of closed porosity. In any case, a temperature of 550 °C, a pressure of 100 MPa, and a dwell time of 60 min were selected.

### 2.6. Sintered Glass Part Characterization

The sintered glass parts were characterized by means of different methods. The final density after sintering or HIP was measured according to Archimedes’ principle by applying a Sartorius YDK01 balance (Sartorius, Göttingen, Germany). Between 2 and 12 samples were considered. The surface appearance and the inner structures of the glass parts were evaluated by SEM. For microscopy, the samples were embedded and then ground with a Saphir 550 (QATM, Mammelzen, Germany). Grinding was carried out in 4 steps with water: First, the samples were ground flat with 46 µm paper and then with 30, 16, and 10 µm paper for 30 s each. Polishing was carried out for 30 min using 6 µm and 3 µm silk cloths with a diamond paste. The SEM measurements were then obtained with a Supra 55 FE–SEM (Zeiss, Oberkochen, Germany) at an accelerating voltage of 10 kV. Additional CT scans of selected samples (Phoenix v tome xs; General Electric, Frankfurt, Germany) provided information on the presence of inner defects like voids or cracks. The accessible spatial resolution was 15 µm (measuring time: 100 ms; voltage: 10 kV, current: 120 µA). Optical transmission measurements were carried out using a UV/Vis spectrometer (SPECORD S 600; Analytikjena, Jena, Germany).

## 3. Results and Discussion

### 3.1. Feedstock Compounding and Melt Flow Behavior

In the following sections, the influence of the binder composition on the compounding process as well as on the rheological behavior will be discussed comprehensively. Extensive feedstock development took place parallel to the injection molding trials in iteration loops to adjust the replication-relevant feedstock properties, such as viscosity for complete mold filling and green body stability for successful demolding. For a better overview, feedstocks with common features will be discussed in the subsections covering a systematic variation in individual binder components.

#### 3.1.1. Influence of the PMMA’s Average Molecular Mass

To cover a wide range of different average molecular masses, feedstock systems 1–4 were prepared with a constant solid load (50 Vol% borosilicate glass), constant PEG type (PEG 8000), constant PEG/PMMA ratio (50:50), and SA amount (4.4 mg/m^2^) (see Table 2). They will be discussed below.

**Figure 2:**
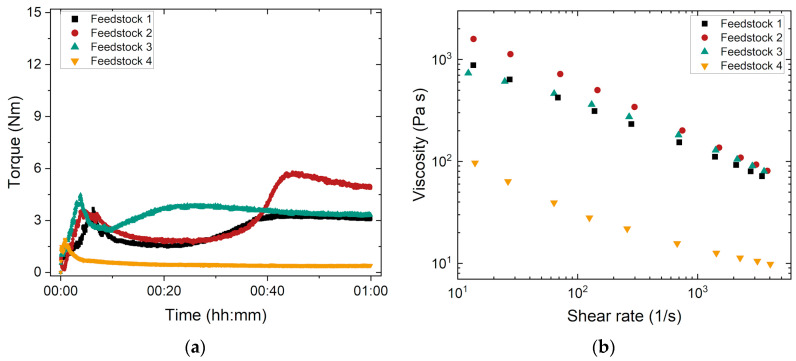
(**a**) Compounding of the feedstocks 1–4 containing different PMMA types (compounding temperature of feedstocks 1–4: 160 °C; (**b**) change in the melt viscosity as a function of the shear rate (measuring temperature: 170 °C).

Thanks to the use of an in-line torque recording mixer–kneader, feedstock homogeneity during compounding could be validated [31] based on the shape and absence of any signal scattering at the end of the stationary state. From previous experience, a final torque of less than 20 Nm ensures a good injection moldability [31]. Figure 2a shows the compounding behavior and Figure 2b shows the change in the melt viscosity versus the shear rate for the four feedstocks listed in Table 2. Feedstock 4 shows the three typical states of a compounding curve as described in the literature (Figure 2a) [31]:

- Filling state: pronounced torque increase caused by filling all materials (PEG, PMMA, SA, and glass powder) into the kneader and pronounced friction between the solid glass particles prior to wetting.
- Mixing state: drop in the torque curve due to the disruption of agglomerates and particle wetting by the different binder components, especially the surfactant.
- Equilibrium (stationary) state with a stable final torque value reflecting a homogeneous feedstock and good moldability.

The other three feedstocks 1–3 exhibited a more complex behavior. After filling, the torque decayed, followed by another torque increase to a stable final value that was higher than that of feedstock 4 containing the PMMA with the very low M_W_. The second torque rise observed may be explained by the morphology and the M_W_ values of the added PMMAs. While PMMA 120k consists of small plates, G77 and G7E are standard pellets with a typical diameter of ~2 mm and a length around 3–4 mm. PMMA 15k is a fine powder that can be fused easily at the elevated compounding temperature. In the case of the other PMMAs, the plates and pellets must be liquefied prior to particle wetting, which explains the delayed equilibrium state. The molecular mass of the used PMMAs has a pronounced impact on the compounding process [32]. The sequence of the final torque value correlates directly with the order of the used M_W_ values of the PMMAs. An increasing M_W_ causes a higher equilibrium torque due to polymer chain entanglement. This higher torque is equivalent to the enhanced inner friction that must be overcome by the mixer–kneader equipment during compounding. Figure 2b shows the related melt viscosity measurement. In all cases, a pronounced pseudoplastic flow can be detected. In accordance with the observation made for compounding, the melt viscosity of feedstock 4 is lower by almost one order of magnitude than the melt viscosities of mixtures 1–3, which can also be attributed to the low M_W_. In agreement with the compounding results, feedstock 2 reaches the highest melt viscosity in the whole shear rate range investigated.

#### 3.1.2. Influence of the PEG’s Average Molecular Mass and Stearic Acid Amount

In the previous subsection, it was shown that the combination of G7E with PEG 8000 and a solid load of 50 Vol% was difficult to compound. G7E is a widely used commercial PMMA type (old tradename Degalan G7E). To reduce material costs, it is recommended to use a standard common polymer. For successful process chain development, the glass filler load was raised up to 60 Vol%, which is helpful in sintering. This solid load increase generally results in a melt viscosity increase [28]. For this reason, the PEG/PMMA ratio was shifted to higher PEG amounts, which causes a viscosity drop and facilitates compounding. To investigate the influence of the PEG’s average molecular mass M_W_ and the amount of stearic acid on compounding as well as on the melt flow behavior, feedstocks 5–10 were used (Table 3). The fraction of the low-cost G7E was kept at a constant solid load of 60 Vol%. The variation in the PEG’s M_W_ was investigated to ensure a good melt viscosity for powder injection molding needed for complete mold filling at moderate temperatures and sufficient mechanical stability during demolding. Another possibility of viscosity adjustment and improved feedstock homogeneity was to choose an appropriate surfactant SA concentration. Initially, a value of 4.4 mg/m^2^ was selected, which had been found to be well-suited for ceramics [18]. It was increased up to 20 mg/m^2^, which corresponds to approximately 3 wt.% of the binder. This high value has been proven to be useful for metals in the literature, especially if the particles are larger and possess a smaller specific surface area [29] compared to the glass filler used here.

**Figure 3:**
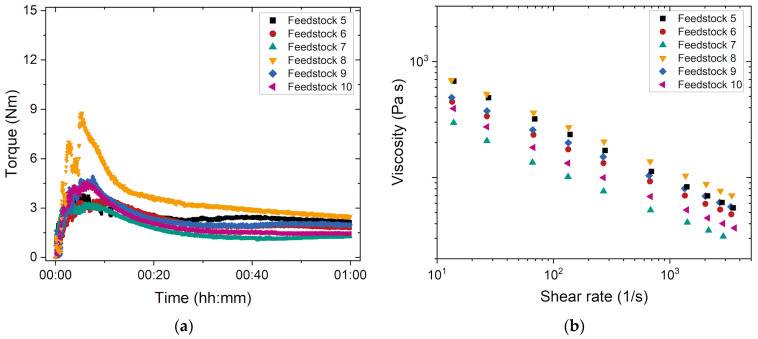
(**a**) Compounding of the feedstocks 5–10 containing different PEG types and SA contents (compounding temperature: 160 °C); (**b**) change in the melt viscosity versus shear rate (measuring temperature: 170 °C).

The significantly higher PEG content in the feedstock suppressed the previously observed (Figure 2a) retarded PMMA melting and the compounding curves with the three typical stages explained earlier were obtained (Figure 3a). As regards binders with identical SA amounts but different PEG types, such as systems 5 and 8, it was found that the binder with the PEG of lower molecular weight produced lower final torque values. When the PEG and SA amounts are the same, an increase in SA also leads to a torque drop. This is also obvious from the melt rheology measurements. With increasing SA concentration, the final torque decreases. The decrease in viscosity as a result of an increasing SA content is not exclusively due to the better wettability of the glass particles with the binder itself, but also to the fact that the feedstock contains less PMMA. In addition, the torque decreases when using a PEG of lower molecular mass. This can be attributed to the shorter, less entangled molecular chains of PEG 8000 compared to PEG 20,000 enabling a better polymer chain sliding at elevated temperatures, which leads to lower viscosity at a given temperature (Figure 3b). The influence of the PEG’s average molecular mass on compounding and melt rheology was also observed in feedstocks containing Ti alloy [28]. The impact of increasing SA amounts on the melt viscosity is more pronounced than that of the PEG’s M_W_ variation. The used PEG as well as the SA content are powerful parameters for the optimization of the injection molding process. As in the previously investigated feedstocks 1–4, a pronounced pseudoplastic melt flow can be observed, which supports injection molding.

#### 3.1.3. Influence of the PEG/PMMA Ratio

**Figure 4:**
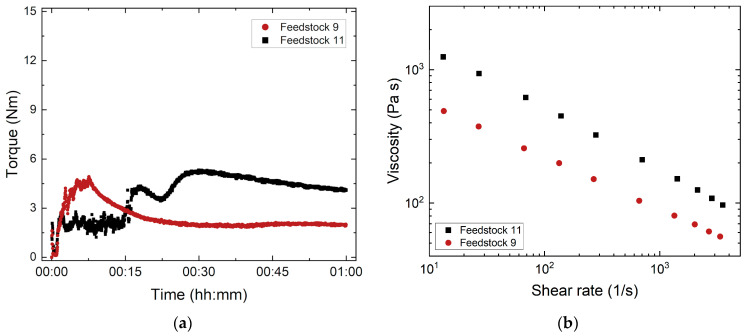
Investigation of feedstocks 9 and 11 with different PEG/PMMA ratios: (**a**) Compounding at 160 °C; (**b**) change in the melt viscosity with shear rate (measuring temperature: 170 °C).

As described in the previous subsection, the variation in the PEG/PMMA ratio allows for an adjustment of the melt viscosity [28]. Increasing PEG amounts reduce the mechanical stability of the green body. It is therefore recommended to use a PEG with a higher M_W_. Based on the binder combination of PEG 20,000/PMMA G7E with an SA content of 10 mg/m^2^ and a solid load of 60 Vol%, the influence of the PEG/PMMA ratio on compounding and melt rheology was studied (Table 4). Figure 4 represents the influence of the PEG/PMMA ratio on torque (a) and melt viscosity (b). The torque curve at the PEG/PMMA ratio of 65:35 shows a typical behavior, while the feedstock with the higher PMMA amount (PEG/PMMA ratio of 50:50) exhibits a non-ideal behavior prior reaching the equilibrium stage. The PMMA needs longer to liquefy, as it is added in the form of large pellets. After that, the torque decreases due to agglomerate wetting, followed by glass agglomerate disruption causing a torque increase. The higher amount of PMMA results in a pronounced equilibrium torque. The same tendency can be found in melt rheology. The feedstock with the lower PMMA amount (PEG/PMMA ratio of 65:35) ensures a significant viscosity drop over the whole shear rate range. Again, a clear pseudoplastic flow can be seen.

#### 3.1.4. Influence of the Solid Load

As a result of the positive outcome of the injection molding trials (see Section 3.2), feedstock 11 was slightly modified by a pronounced SA increase up to 25 mg/m^2^ to guarantee simple compounding as well as good and reliable mold filling. This feedstock was denoted feedstock 12 (Table 5) and used as the starting point for a further increase in the solid load up to 70 Vol% (feedstocks 13–16).

**Figure 5:**
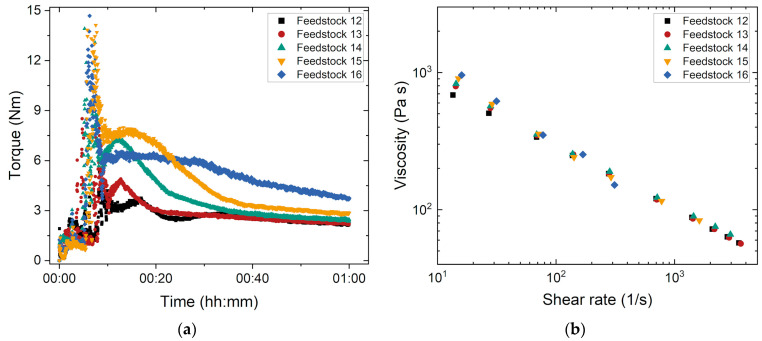
Investigation of feedstocks 12–16 with increasing solid loads: (**a**) Compounding at 160 °C; (**b**) change in the melt viscosity with shear rate (measuring temperature: 170 °C).

Figure 5 shows the influence of powder load on the torque and melt viscosity of feedstocks 12–16. As expected, the equilibrium torque increases non-linearly with the increasing powder load. Between 60 and 65 Vol% solid loads, the torque gain is small. When exceeding 68 Vol%, however, a pronounced rise can be measured. The torque of feedstock 16 (70 Vol% load) shows no equilibrium phase after one hour. It is assumed that the feedstock is not yet completely homogeneous, and the particles are not completely wetted (Figure 5a). The torque–time curve clearly shows the limitation of the maximum processable solid load in this binder system. Irrespective of the solid load, all feedstocks show a pronounced pseudoplastic flow (Figure 5b). The influence of the filler content becomes particularly evident at lower shear rates. With an increasing shear rate, the different amounts of glass filler cause no major viscosity differences. In general, the feedstocks with a solid load of 68 and 70 Vol% reach the highest viscosities over the entire shear rate range.

#### 3.1.5. Influence of the PMMA’s Average Molecular Mass–Revisited

**Figure 6:**
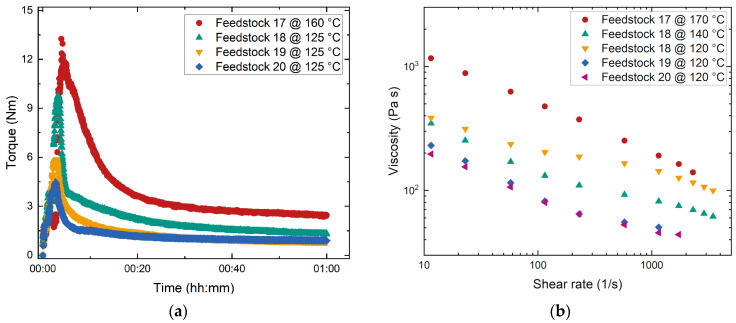
Investigation of feedstocks 17–20 containing different PMMAs at an SA content of 25 mg/m^2^: (**a**) Compounding; (**b**) change in melt viscosity with shear rate.

Following the results of the feedstocks 12–16 with the high SA content and the promising compounding and melt viscosity properties, the impact of the average molecular mass on the feedstock properties were investigated again using the two PMMAs from Sigma (Table 1) at a constant solid load of 60 Vol%. In addition, the influence of the PEG type was studied (Table 6). Figure 6a shows the compounding curves. Due to the high M_W_ of the PMMA Sigma 120k binder, the mixing temperature was set to 160 °C (feedstock 17). For the Sigma 15k-based feedstocks (feedstocks 18–20), it was possible to lower the mixing temperature to 125 °C. All Sigma 15k-based feedstocks reached the equilibrium state quite quickly. Substitution of PEG 20,000 by PEGs with lower M_W_ values further accelerated the process of reaching the equilibrium state. The same general trend is obvious from the melt viscosity experiment shown in Figure 6b. The reduction in the PEG’s M_W_ in the Sigma 15k-based mixtures from 20,000 down to 8000 caused a pronounced viscosity drop. All feedstocks exhibited a pseudoplastic flow behavior.

### 3.2. Injection Molding

In general, all prepared feedstocks were suitable for injection molding. During part production by GIM, feedstocks 5–10 exhibited almost the same behavior and showed good molding properties. The main differences occurred during cooling time and demolding. For the combination of PEG 8000 and PMMA G7E, the cooling time for demolding of a warpage-free part was between 2 and 3 min, which is very long for such a small part. When the mold is opened prematurely, mechanical stability of the component is not guaranteed and warpage occurs. To reduce the cooling time, we decided to use PEG 20,000 and the PEG to PMMA ratio was set to 50:50 to enhance green body stability. This binder composition, however, led to a viscosity increase. Hence, we looked for a compromise between feedstocks 9 and 11, which both had the low viscosity necessary in combination with an enhanced mechanical stability during demolding after a more acceptable cooling time of 30 s. This new feedstock 12 consisted of PEG 20,000 and PMMA G7E at a ratio of 50:50 with an increased SA content of 25 mg/m^2^ and was chosen for further investigation. Due to the composition changes to achieve good mold filling and a high strength of the green body during demolding, a stable and robust injection molding process was possible. Even the feedstock with the elevated solid load of 65 Vol% (feedstock 14) could be processed without any mold filling and demolding difficulties. When exceeding a glass content of 65 Vol%, mold filling started to be problematic. The high SA content of 25 mg/m^2^ in feedstocks 17–20 also ensured reliable injection molding. A comprehensive overview of the injection molding trials producing the best part results in terms of complete mold filling and easy demolding is given in Table 7.

### 3.3. Debinding

After replication and prior to densification by sintering, all organic binder moieties must be removed by dissolution, temperature-based decomposition, or a combination of both processes, which has been adjusted to the binder components with low- and high-M_W_ polymers. The combined process is common in PIM due to pore formation during solvent pre-debinding, which allows for the diffusion of degraded polymer fragments out of the bulk material without damaging the shape of the samples [33,34].

#### 3.3.1. Liquid Pre-Debinding

The PEG/PMMA binder allows for the eco-friendly use of water as a solvent for the liquid pre-debinding step [28]. During liquid debinding, time and temperature are the two key parameters relevant to PEG dissolution. Using feedstock 12 as an example, Figure 6 shows the PEG removal with time and temperature. The values obtained for feedstocks with higher solid loads at a fixed debinding time are also indicated.

**Figure 7:**
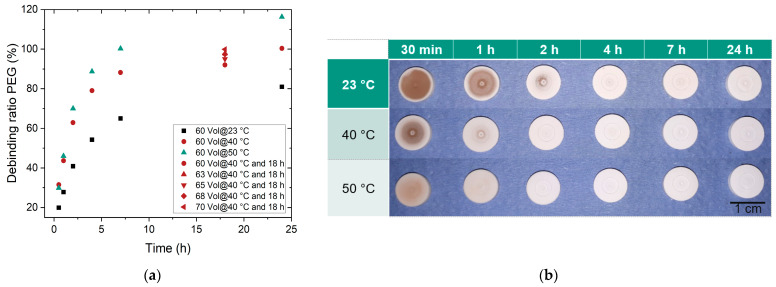
Liquid pre-debinding in water when applying feedstock 12: (**a**) Debinded normalized PEG amount (%). In addition, values after a debinding time of 16 h are added for feedstocks 12–16. (**b**) Sample images reflecting the qualitative state of liquid debinding: first row: 23 °C; second row 40 °C; third row: 50 °C debinding temperature.

The degree of debinding increased strongly in the beginning due to the high concentration gradient of PEG between the green body and water. Then, it leveled off. A diffusion process, debinding was accelerated with the increasing temperature, which agreed with previous investigations [32,35]. A theoretical debinding degree of 100% at 50 °C was already reached after approx. 7 h, whereas approx. 80% of the PEG only had disappeared after 24 h at 23 °C (room temperature). At a water temperature of 50 °C, not only the water-soluble PEG, but also fractions of the partially soluble SA were removed within 24 h. Figure 7b shows that the PEG dissolved along the surface. In the further course of this work, the components were subjected to liquid pre-debinding for 16 h at 40 °C. A sufficient degree of debinding of more than 90% was achieved at a moderate processing time. The debinding degrees of feedstocks 12–16 at 18 h and 40 °C are also shown in Figure 7a. With increasing powder load, the debinding degree increased as well.

**Figure 8:**
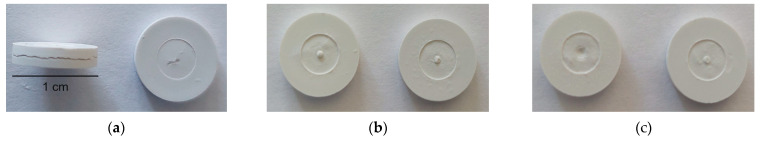
Images of the pre-debinded test samples of 10 mm in diameter and 2 mm in thickness: (**a**) feedstock 18; (**b**) feedstock 19; (**c**) feedstock 20.

The standard liquid pre-debinding program covering a period of 16 h at 40 °C did not work for feedstock 18, because of crack formation (Figure 8a). This was attributed to the fact that the molecular mass of PEG was higher than that of PMMA. Normally, this should be the other way around. The short PMMA chains cannot act as a backbone polymer to ensure a certain mechanical stability in case of solvent-induced polymer swelling, for instance. For this reason, the PEG 20,000 used in feedstock 18 was replaced by PEG 8000 in feedstock 19 and by PEG 4000 in feedstock 20. After liquid pre-debinding, the parts made of feedstock 19 (Figure 8b) showed no visible cracks, but there were small bubbles on the surface. Further reduction in the PEG’s M_W_ (feedstock 20) led to a defect-free component after liquid pre-debinding (Figure 8c). The PEG swells slightly when dissolved in water. This spatial expansion cannot be sufficiently cushioned by the low-molecular PMMA. As the molecular weight of PEG decreases, spatial expansion decreases, and cracking is prevented.

#### 3.3.2. Thermal Debinding

**Figure 9:**
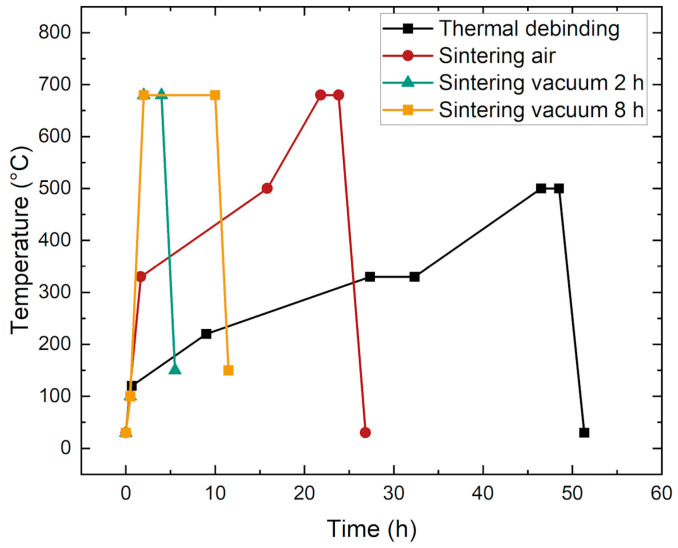
Temperature programs used for thermal debinding and sintering.

Thermal debinding must be performed as slowly as possible, because thermal decomposition of the organic feedstock components is accompanied by a pronounced volume expansion due to the generation of gaseous products, which can cause cracks or total disintegration of the part. Figure 9 shows the thermal debinding program with very slow heating rates, especially in the temperature range from 120 °C to 330 °C, according to the thermal behavior of the major organic components PEG and PMMA. The debinding program was adapted from [32]. Major decomposition started around 220 °C, which led to very small heating rates and the long dwell time at 330 °C. This thermal debinding program was used for all samples, irrespective of whether or not there was a solvent-assisted pre-debinding step.

### 3.4. Sintering Process Development

**Figure 10:**
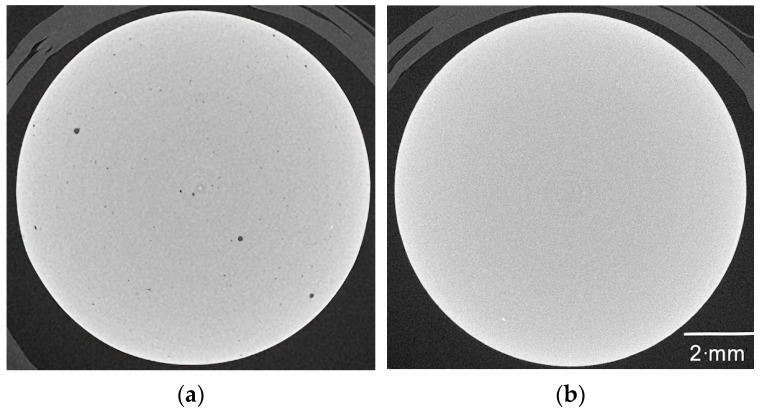
CT scans of sintered glass samples when applying feedstock 5 for replication: (**a**) only thermal debinding; (**b**) combination of liquid pre-debinding and thermal debinding.

Sintering was performed in two different ways, either in air or under vacuum. The different temperature programs are also presented in Figure 9. The reason for the different temperature programs is the HTF vacuum oven that was used, which could not handle small heating rates. Hence, the smallest possible rates were used. To find out whether liquid pre-debinding was necessary or not, sintered samples with and without pre-debinding were non-destructively characterized by CT scans (Figure 10). Figure 10a shows the sample after thermal debinding and subsequent sintering in air. Figure 10b presents the sample after pre-debinding in water, thermal debinding, and sintering in air. As can be seen, the sintered and thermally debinded component has dark spots inside. In contrast to this, the component subjected to a preceding aqueous pre-debinding step does not exhibit dark spots. The dark areas in the CT scan are areas where the density is lower than in the light areas. Since no binder is present after sintering, this must be air. Consequently, the dark areas are equivalent to voids. This is also confirmed by the sinter densities of the components measured using Archimedes’ principle. The only thermally debinded component has a sinter density of 96.9%, while the two-step debinded sample reaches 98.7% of the theoretical value. Consequently, it is strongly recommended to apply the two-step debinding procedure.

**Figure 11:**
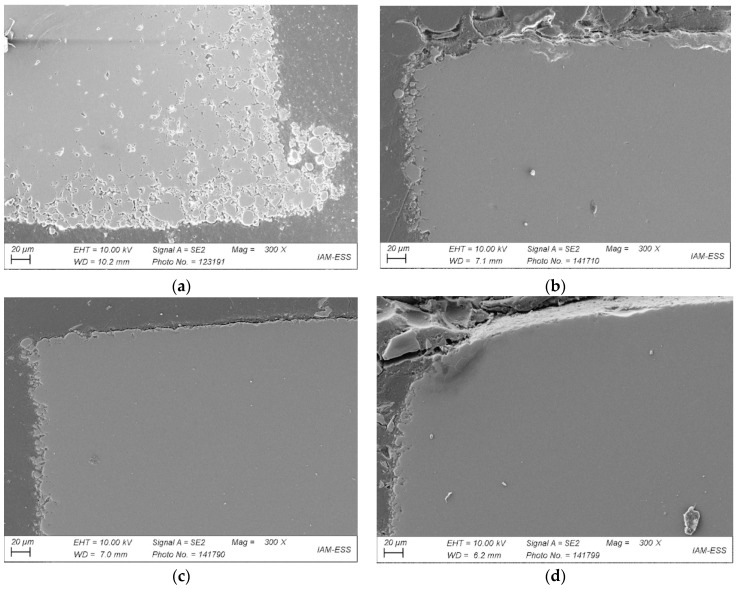
SEMs of sintered samples based on feedstock 12: (**a**) 2 h sintering in air; (**b**) 2 h under vacuum; (**c**) dwell time of 2 h under vacuum plus HIP at 550 °C; (**d**) dwell time of 8 h under vacuum plus HIP at 550 °C.

**Figure 12:**
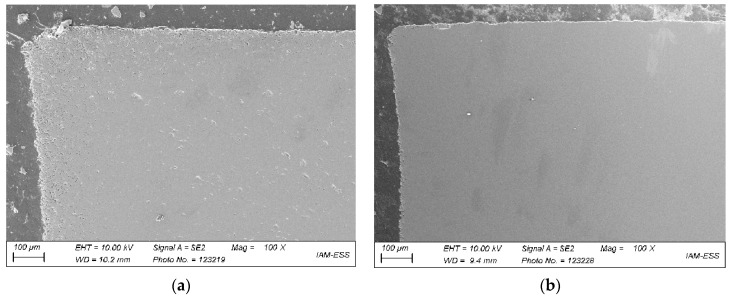
SEMs of sintered samples based on feedstock 14: (**a**) 2 h sintering in air; (**b**) 2 h under vacuum.

The influence of the two different sintering conditions (Figure 9)—air or vacuum—can be seen in Figure 11a,b, both of which were taken after applying the same temperature program. All parts were originally made of feedstock 12. Two main differences can be seen: first, vacuum sintering improved the quality of the outer contour and reduced open porosity; second, no voids can be detected in the bulk part. The higher heating rates did not adversely affect the part quality. As a result, processing costs were reduced. In addition, the influence of hot isostatic pressing (HIP) on part quality after vacuum sintering was investigated (Figure 11c,d). Figure 11b–d show several anomalies. For all three components sintered under vacuum, open porosity is obvious at the edges. The porosity decreases with increasing sintering time and subsequent HIP. Figure 11b shows the part sintered at 680 °C in vacuum after 2 h. Figure 11c represents the part sintered for 2 h at 680 °C in vacuum with subsequent HIP at 550 °C and 100 MPa for one hour. Figure 11d shows the microstructure after vacuum sintering with a dwell time of 8 h at 680 °C and subsequent HIP. With increasing sintering time and additional HIP, sintering warpage increases. In general, vacuum sintering without HIP is sufficient for a pronounced void reduction. As regards further part quality improvement, the impact of the solid load in the feedstock on processing and on the sintered part can be seen in Figure 12. Again, more voids are found in the air-sintered sample (Figure 12a) compared to the sample sintered under vacuum (Figure 12b). Due to the small effect of HIP on the sample quality described above, this additional process step was omitted.

**Figure 13:**
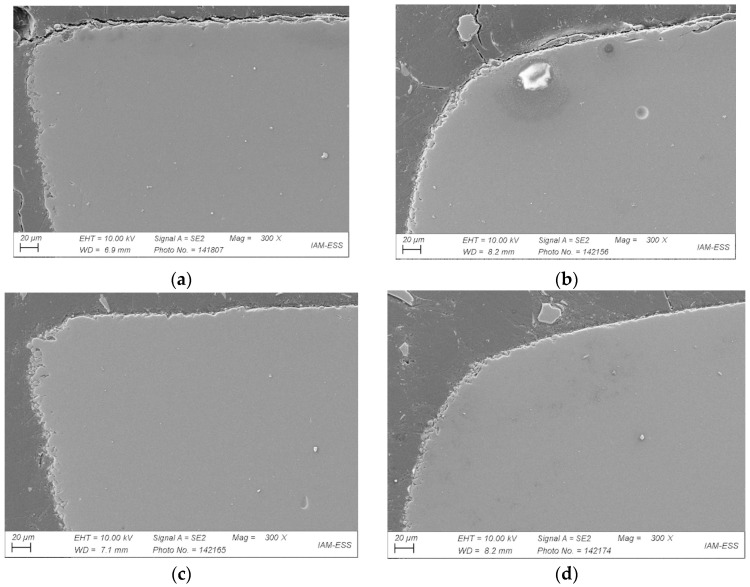
SEMs of sintered samples based on feedstock 17: (**a**) 2 h vacuum sintering; (**b**) 8 h vacuum sintering; (**c**) 2 h vacuum sintering plus HIP; (**d**) 8 h vacuum sintering plus HIP.

The influence of the PMMA’s M_W_ in combination with high SA amounts on processing was investigated in feedstock 17. Figure 13 shows micrographs of four parts fabricated from this PMMA Sigma 120k-based feedstock. Figure 13a shows the microstructure after 2 h of vacuum sintering, Figure 13b after 8 h of vacuum sintering. Figure 13c presents the sample after 2 h of vacuum sintering and additional HIP. Figure 13d shows the sample after 8 h of vacuum sintering. No defects can be seen in all these four components. Their edges and shapes, however, are different. Open porosity decreases with increasing sintering time.

**Figure 14:**
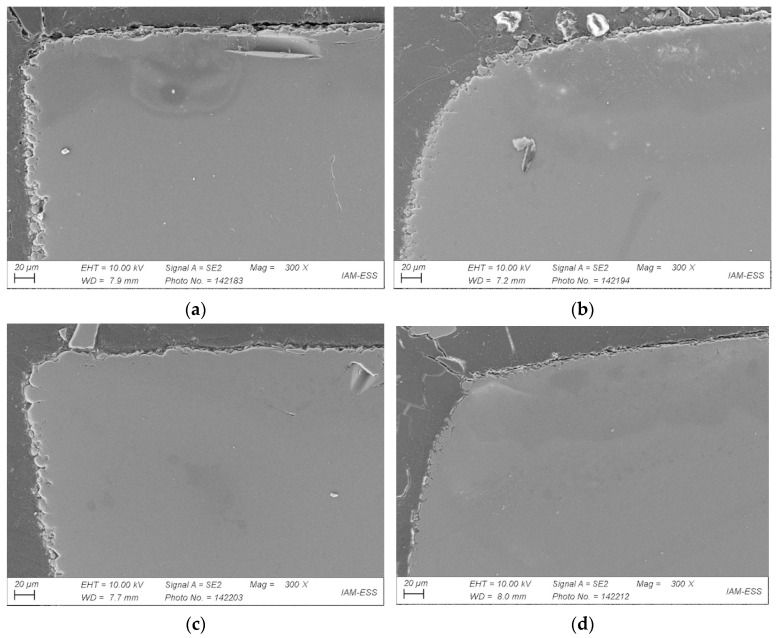
SEMs of sintered samples based on feedstock 20: (**a**) 2 h vacuum sintering; (**b**) 8 h vacuum sintering; (**c**) 2 h vacuum sintering plus HIP; (**d**) 8 h vacuum sintering plus HIP.

In addition, HIP treatment was found to lead to a slight reduction in open porosity. The distortion at the corners of the component increased significantly with increasing sintering time. The HIP treatment hardly had any influence on this. In conclusion, it can be stated that the extension of the sintering time has a greater influence on open porosity reduction than the additional HIP process. However, higher warpage occurs, as is obvious from Figure 13b,d. To finalize the investigations of the impact of feedstock composition on processing and the appearance of the resulting part, feedstock 20 containing the low-molecular-mass PMMA 15k was studied using the same process parameters (Figure 14a–d). As in the previous examples (feedstock 17, Figure 13), an increasing sintering time reduced open porosity, but caused a pronounced rounding off due to surface minimization, which is the driving force of sintering (Figure 14b,d). Again, additional HIP was found to result in a minor improvement only.

### 3.5. Influence of Feedstock Composition, Debinding, and Sintering on Part Density

An important criterion in the critical validation of the GIM process chain is the final sinter density. Table 8 shows the resulting sinter densities achieved for all feedstocks, which were completely processed. The influence of the debinding process is presented as well. The data were measured before the samples were ground for SEM. Table 8 clearly shows that the combination of liquid pre-debinding and thermal debinding always leads to higher density values. Contrary to the expectations, the increase in solid load in feedstocks 13–16 compared to feedstock 12 did not cause any remarkable density increase. Density remained almost constant at a high level.

Hence, the higher effort associated with the compounding and injection molding of feedstock loadings beyond 60 Vol% is not paid off by a higher sinter density. A filler load of 60 Vol% is sufficient to achieve the best sinter results. More details on the sinter parameters and the resulting density outcomes are obvious from Table 9, which summarizes the impact of the vacuum sinter time and additional HIP treatment. For all three investigated feedstocks (12, 17, 20), very good sinter densities of better than 99% theoretical density can be achieved when the samples are sintered under vacuum for 2 h at the maximum temperature. These values are in the same range as those described in [30]. Neither a further increase in sinter time nor the use of HIP improves the sinter values in a relevant way. On the contrary, a longer sinter time leads to sample deformation.

### 3.6. Optical Properties

**Figure 15:**
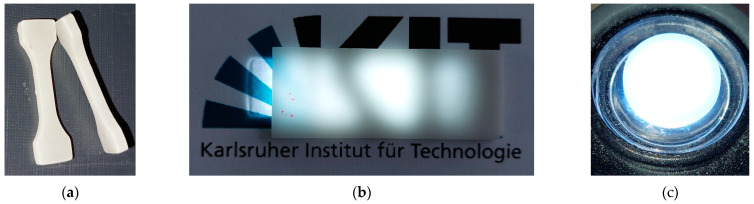
Sintered glass samples: (**a**) micro tensile specimen; (**b**) sintered plate; (**c**) sintered round test sample (diameter 0.82 cm).

**Figure 16:**
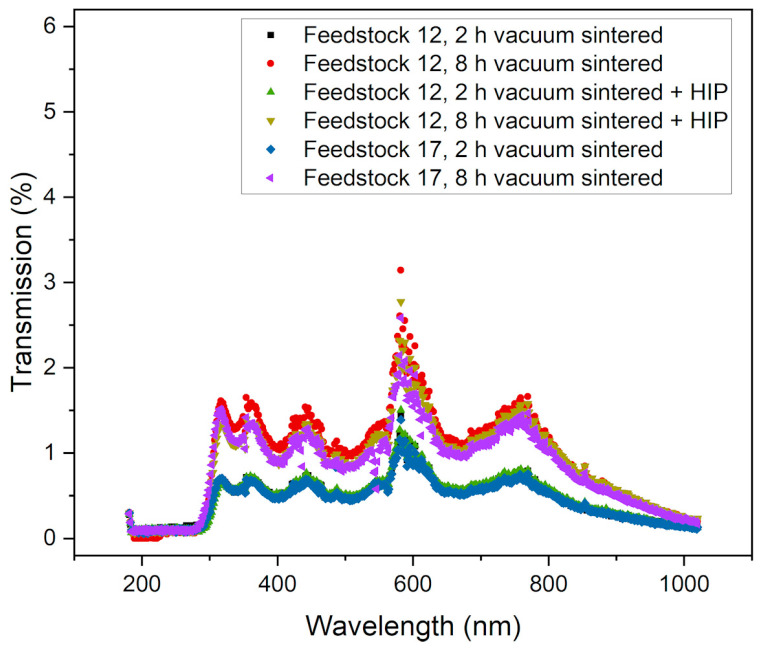
Optical transmittance spectra of samples sintered under different processing conditions.

It is evident from the previous sections that even under optimized sinter conditions, surface layers possess a certain open porosity and defects, which reduce optical transparency and allow for a certain translucency only when illuminated from the back. Figure 15a shows a sintered micro tensile specimen designed for mechanical characterization. A pronounced surface reflection due to light scattering can be seen. Figure 15b represents a larger sintered plate (thickness 3.7 mm) and Figure 15c represents a 1.8 mm thick sintered round test sample as used in the previous micrographs Both are illuminated from the back by LED white light and show a certain translucency. The samples in Figure 15a,c were originally made of feedstock 12; the sample shown in Figure 15b was based on feedstock 18. Optical transmission spectra were recorded for samples with different process histories, especially sinter parameters (Figure 16). In the visible range (380–780 nm) of the investigated wavelengths, all samples possess a small optical transmittance between 0.5 and 1.5%, with a maximum of up to 3% around 600 nm. A clear correlation between sample history and transmittance cannot be detected. The best values were obtained for feedstock 12 with a vacuum sintering time of 8 h with and without HIP.

## 4. Conclusions

The most important results of the tests reported are listed below:

1. The glass injection molding process chain was evaluated systematically, from feedstock development to molding, to debinding, to sintering.
2. In the first process step, feedstock development using the given binder components PEG, PMMA, and SA, the average molecular masses of PEG and PMMA, their ratios, and the SA content were varied to enable simple, fast, and reliable compounding as well as good molding. During replication, good mold filling as well as defect-free stable demolding were ensured by selecting suitable feedstock compositions. As regards the debinding procedure, it was found that a combination of liquid pre-debinding and thermal treatment was recommended. This could be verified after the final sinter process. In this step, vacuum sintering is also favorable to achieve the highest sinter densities. Sinter densities of around 99–100% of theoretical density could be achieved.
3. An increase in the initial feedstock’s solid load does not result in any improvement in the final sinter densities and part appearance. This also holds when an additional thermal post-treatment by HIP takes place.
4. Suitable feedstock systems with 60 Vol% glass filler, 25 mg/m^2^ SA, and PEG as well as PMMA having different average molecular weights in combination with two-step debinding and vacuum sintering can be recommended for further investigations.
5. A certain translucency was measured, with optical transmission values reaching up to 3% in the visible range.
6. The comprehensive investigations allow for a clear correlation between the feedstock composition and the influence of each individual binder component on compounding, molding, debinding, and sintering.

## 5. Outlook

Future work should focus on a precise evaluation of mechanical properties as a function of the feedstock composition and the realization of functional devices like ceramic microreactors with integrated electrical sensors. In addition, studies should be extended to additive manufacturing applying MEX methods.

## Figures and Tables

**Table 1:** Used binder components, their functions, and the corresponding suppliers.

Binder Component	Function	Supplier
PEG 4000	Plasticizer	Carl Roth GmbH + Co., KG, Karlsruhe, Germany
PEG 8000	Plasticizer	Carl Roth GmbH + Co., KG, Karlsruhe, Germany
PEG 20000	Plasticizer	Carl Roth GmbH + Co., KG Karlsruhe, Germany
PMMA G77	Backbone Polymer	Quinn Plastics GmbH, Mainz, Germany
PMMA G7E	Backbone Polymer	Röhm, Darmstadt, Germany
PMMA 120k	Backbone Polymer	Sigma-Aldrich, Taufkirchen, Germany
PMMA 15k	Backbone Polymer	Sigma-Aldrich, Taufkirchen, Germany
SA	Surfactant/Release agent	Carl Roth GmbH + Co., KG, Karlsruhe, Germany

**Table 2:** Overview of investigated feedstock systems with different PMMA types.

Feedstock ID	PMMA Type	Average Molecular Mass M_W_ (g/mol)
1	G77	88,000 [33]
2	G7E	159,000 [28]
3	Sigma 120k	120,000 ^1^
4	Sigma 15k	15,000 ^1^

^1^ Values taken from vendors’ data sheets.

**Table 3:** Feedstocks with G7E as PMMA, different PEG types, and variable SA contents at a solid load of 60 Vol% glass filler.

Feedstock ID	PEG Type	PEG/PMMA Ratio	SA Conc. (mg/m^2^)
5	8000 ^1^	65:35	4.4
6	8000 ^1^	65:35	10
7	8000 ^1^	65:35	20
8	20,000 ^1^	65:35	4.4
9	20,000 ^1^	65:35	10
10	20,000 ^1^	65:35	20

^1^ The given number is equivalent to the average molecular mass M_W_. All values are taken from vendors’ data sheets.

**Table 4:** Overview of the used feedstocks with PEG 20,000 and PMMA G7E, constant SA contents, and solid loads (60 Vol%), but different PEG/PMMA ratios.

Feedstock ID	PEG Type	PMMA Type	PEG/PMMA Ratio	SA Conc. (mg/m^2^)
9	20,000	G7E	65:35	10
11	20,000	G7E	50:50	10

**Table 5:** Overview of the used feedstocks with increasing solid loads.

Feedstock ID	Solid Load (Vol%)	PEG Type	PMMA Type	PEG/PMMA Ratio	SA Conc. (mg/m^2^)
12	60	20,000	G7E	50:50	25
13	63	20,000	G7E	50:50	25
14	65	20,000	G7E	50:50	25
15	68	20,000	G7E	50:50	25
16	70	20,000	G7E	50:50	25

**Table 6:** Overview of the feedstock systems investigated with two different PMMA types and high SA concentration.

Feedstock ID	Solid Load (Vol%)	PEG Type	PMMA Type	PEG/PMMA Ratio	SA Conc. (mg/m^2^)
17	60	20,000	Sigma 120k	50:50	25
18	60	20,000	Sigma 15k	50:50	25
19	60	8000	Sigma 15k	50:50	25
20	60	4000	Sigma 15k	50:50	25

**Table 7:** Injection molding parameters resulting in best molding qualities.

Feedstock System	Applied Feedstocks	Melt Injection Temperature (°C)	Injection Speed (mm/s)	Cooling Time (s)
PMMA G77 based	1	170	50	30
PMMA G7E-based	2, 11–16	170	50	30
PMMA G7E-based	5–10	170	50	120–180
PMMA 120k-based	3, 17	160–170	50–65	20–30
PMMA 15k-based	18–20	105–120	50–65	20–30

**Table 8:** Measured sinter densities of the feedstocks after debinding and 2 h sintering at maximum temperature in air.

Feedstock ID	Debinding Method	Sinter Density (% th.)
2	thermal	95.7
2	liquid, thermal	98.8
3	thermal	92.3
3	liquid, thermal	98.1
5	thermal	96.9
5	liquid, thermal	98.7
6	thermal	97.2
6	liquid, thermal	98.9
7	thermal	97.3
7	liquid, thermal	98.8
9	thermal	97.3
9	liquid, thermal	98.9
10	thermal	97.2
10	liquid, thermal	99.0
11	thermal	96.9
11	liquid, thermal	98.9
12	thermal	96.8
12	liquid, thermal	99.1
13	liquid, thermal	99.0
14	liquid, thermal	99.1
15	liquid, thermal	98.7
16	liquid, thermal	98.7
17	liquid, thermal	n.a., see Table 9
18	thermal	98.4
20	liquid, thermal	n.a., see Table 9

n.a. stands for not available.

**Table 9:** Measured sinter densities of feedstocks 12, 17, and 20 as a function of the sinter conditions (all cases: liquid pre-debinding, thermal debinding, and vacuum sintering).

Feedstock ID	Sintering Method	Sinter Density (% th.)
12	2 h, 680 °C, vacuum	99.8 ± 0.1
12	2 h, 680 °C, vacuum, 1 h, 550 °C, HIP	100.4 ± 0.03
12	8 h, 680 °C, vacuum	99.6 ± 0.04
12	8 h, 680 °C, vacuum, 1 h, 550 °C, HIP	100.3 ± 0.06
17	2 h, 680 °C, vacuum	99.8 ± 0.05
17	2 h, 680 °C, vacuum, 1 h, 550 °C, HIP	100.4 ± 0.04
17	8 h, 680 °C, vacuum	99.5 ± 0.09
17	8 h, 680 °C, vacuum, 1 h, 550 °C, HIP	100.3 ± 0.06
20	2 h, 680 °C, vacuum	99.4 ± 0.3
20	2 h, 680 °C, vacuum, 1 h, 550 °C, HIP	100.1 ± 0.06
20	8 h, 680 °C, vacuum	99.5 ± 0.07
20	8 h, 680 °C, vacuum, 1 h, 550 °C, HIP	100.3 ± 0.08

## Data Availability

Data are contained within the article.
